# Pediatric Nasal Lobular Capillary Hemangioma

**DOI:** 10.1155/2012/769630

**Published:** 2012-08-05

**Authors:** Jordan M. Virbalas, John P. Bent, Sanjay R. Parikh

**Affiliations:** ^1^Department of Otorhinolaryngology-Head & Neck Surgery, Albert Einstein College of Medicine, 3400 Bainbridge Avenue, 3rd Floor, Bronx, NY 10467, USA; ^2^Department of Otorhinolaryngology-Head & Neck Surgery and Children's Hospital at Montefiore, Albert Einstein College of Medicine, Bronx, NY 10467, USA; ^3^Department of Otorhinolaryngology-Head & Neck Surgery and Seattle Children's Hospital, University of Washington School of Medicine, Seattle, WA 98195-6340, USA

## Abstract

*Background.* LCH is a benign vascular growth of the skin and mucous membranes commonly affecting the head and neck. Since it was first described in the nineteenth century, this entity has been variously known as “human botryomycosis” and “pyogenic granuloma.” The shifting nomenclature reflects an evolving understanding of the underlying pathogenesis. We review the histopathology of and current epidemiological data pertaining to LCH which suggests that the development of these lesions may involve a hyperactive inflammatory response influenced by endocrine factors. We report two new cases of pediatric lobular capillary hemangioma (LCH) of the nasal cavity and review current theories regarding the etiology, diagnosis, and treatment of nasal LCH. *Methods*. Retrospective case series. *Case Series*. Two adolescent females presented with symptoms of recurrent epistaxis, nasal obstruction, and epiphora. Both patients underwent computed tomography imaging and biopsy of their intranasal mass. The tumors were excised using image-guided transnasal endoscopic technique. Seven other cases of nasal LCH have been reported to date in the pediatric population. *Conclusion*. Nasal LCH is a rare cause of an intranasal mass and is associated with unilateral epistaxis, nasal obstruction, and epiphora. We advocate for image-guided endoscopic excision of LCH in the adolescent population.

## 1. Introduction

Lobular capillary hemangioma (LCH) is a benign vascular growth of the skin and mucous membranes commonly affecting the head and neck [1, 2]. The evolving terminology associated with this lesion reflects a change in the understanding of its etiology. In 1897, Poncet and Dor published the first report of “pea to nut-sized” vascular tumors on the fingers and arms of four patients [3]. The authors referred to this condition as “human botryomycosis,” speculating that the lesions were secondary to a fungal infection thought to cause morphologically similar lesions in horses. In 1904, Hartzell coined the term “pyogenic granuloma” to describe these lesions which he presumed to be granulation tissue arising in response to a bacterial infection [4]. In 1980, Mills et al., noting the paucity of evidence to support an infectious origin, proposed the term “lobular capillary hemangioma” derived from the characteristic microscopic features of this tumor [1].

The etiology of LCH remains unknown, though there is some evidence to support both trauma and hormonal influences. The relative frequency of LCH developing at the anterior nasal septum and the anterior aspect of the inferior turbinate, as well as the increased incidence of LCH in recurrent nose pickers or those with a history of nasal packing lends credence, give to the belief that local trauma plays arole in the genesis of LCH [5, 6]. Many theorize that these lesions represent an overgrowth of granulation tissue produced by a hyperactive inflammatory response [5, 7, 8].However, a retrospective study of 112 patients by Pagliai and Cohen found a history of trauma in only 5 patients (4.5%) with clinically diagnosed LCH [7]. Further investigation of suspected inciting sources, including insect bites, hemangiomas, dermatologic conditions, and telangiectasias, revealed that 76.8% of patients had no history of these various traumatic, dermatologic, and vascular pathologies thought to be associated with LCH. Other plausible etiologies that have been proposed include viral oncogenes, microscopic arteriovenous malformations, and overproduction of angiogenic growth factors [6].

There is a well-established relationship between LCH and pregnancy. LCH commonly occurs in women who are pregnant and those who use oral contraceptives [9, 10]. These lesions regress after delivery, implicating a role of hormones in the growth of LCH [9, 11, 12].

Though LCH frequently presents in the head and neck, it rarely occurs in the nasal cavity [1, 2, 9, 11, 13].There are only nine reports describing cases in the pediatric population (Table 1).We present two cases of LCH occurring in the nasal cavities of female adolescents. 

## 2. Description of Case Series

### 2.1. Case 1

A 16-year-old female presented with four months of recurrent right-sided epistaxis, nasal obstruction, and epiphora.The patient's past medical history included asthma and mild eczema.She denied oral contraceptive use and prior sexual activity.

**Figure 1:**
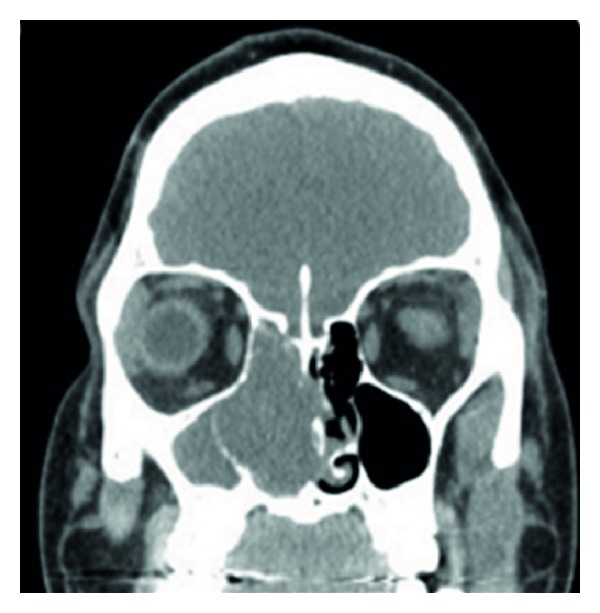
A noncontrast head CT revealing an LCH extending from the right maxillary and ethmoid sinuses to the right nasal choana.

On nasal endoscopy, a pedunculated, grossly vascular tumor was visualized arising from the lateral surface of the middle turbinate.A noncontrast head CT demonstrated a soft tissue mass extending from the right maxillary and ethmoid sinuses to the right nasal choana (Figure 1).There was no bony destruction evident on CT.

Biopsy of the intranasal mass was performed in the office with no significant bleeding.The histopathology demonstrated a dense network of capillary-sized vessels consistent with LCH.The biopsy revealed acutely and dramatically inflamed granulation tissue.The presence of necrosis and squamous metaplasia was thought to suggest trauma with reactive changes.

The patient was treated with image-guided endoscopic excision of the right nasal mass arising from the right middle meatus.Her surgery and recovery were uncomplicated and no recurrence has been noted six months postoperatively.The final pathology was read as lobular capillary hemangioma.

### 2.2. Case 2

**Figure 2:**
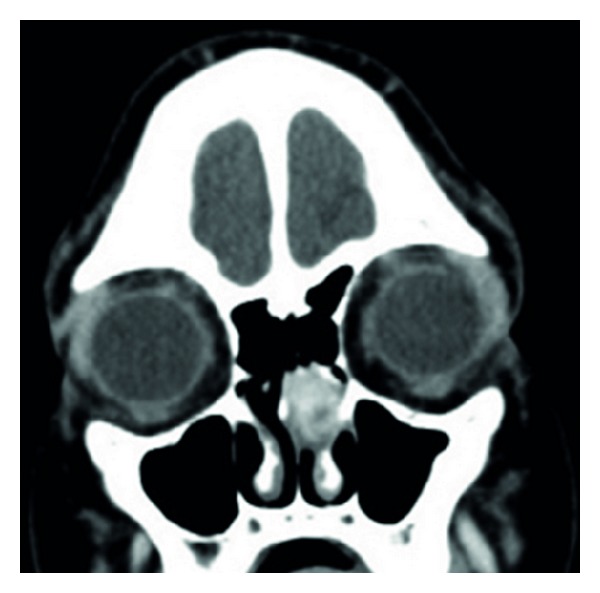
A head CT with contrast demonstrating an LCH in the left nasal cavity.

A 12-year-old female presented with three months of recurrent, left-sided, epistaxis and nasal obstruction.On nasal endoscopy, the patient was noted to have a mass filling the left middle meatus.A head CT with contrast demonstrated a mass in the left nasal cavity (Figure 2).The mass was biopsied in the office without significant bleeding after the procedure. Pathologic evaluation revealed proliferating small vessels and spindled cells in a myxoid stroma.These vessels assumed a lobular architecture in some foci.The pathology was read as polypoid capillary hemangioma.

**Figure 3:**
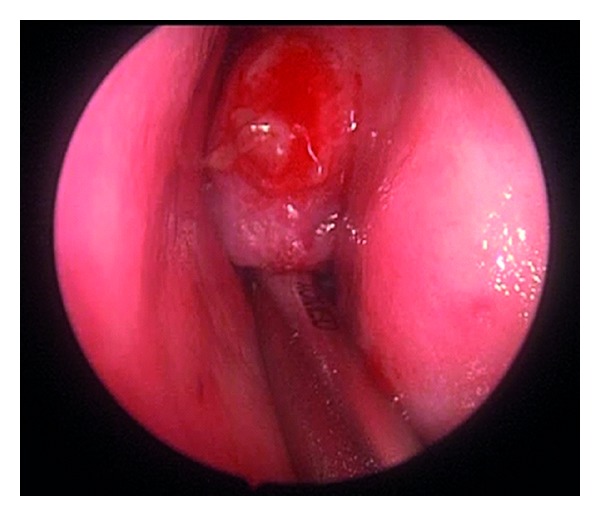
Intraoperative image of an LCH arising from the left lateral nasal wall.

The patient underwent an image-guided transnasal endoscopic resection of this mass (Figure 3).The mass was noted to arise from the left lateral nasal wall anterior to the middle turbinate.Her surgery and recovery were uncomplicated and no recurrence has been noted to date, more than three years after her surgery.The final pathology was capillary hemangioma.

## 3. Discussion

LCH occurs at any age, but most commonly develops in the third to fifth decade of life [6]. In adults, LCH occurs in as many as 5% of all pregnant women [6, 9, 12]. Pagliai and Cohen found that, among 115 children presenting with LCH, more than 60% were male [7]. The same study identified the most common location of these lesions in children to be the head and neck (76.9%). Among those patients with head and neck LCH, the majority had lesions on the skin (36.1% on the cheek, 12% on the forehead, 9.6% on the scalp). Other studies report the frequent appearance of these lesions on the gingiva, lips, tongue, buccal mucosa, and rarely in the nasal cavity [1, 2, 5]. When the nasal mucosa is affected, the lesions typically involve the anterior portion of the inferior turbinate or Little's area on the anterior nasal septum [5, 20, 21]. The relative increase in incidence at these sensitive sites nearest the nares lends support to the theory that local trauma may precede the development of LCH [6].

Patients with LCH of the nasal cavity commonly present with epistaxis, nasal obstruction, epiphora, or purulent rhinorrhea [6, 8]. The diagnosis is often made clinically, based on a characteristic history of a rapidly enlarging, pedunculated mass with intermittent epistaxis [7]. 

Radiographic studies are frequently used to diagnose an intranasal mass.CT can be important in determining the presence of bony destruction by a rapidly growing intranasal mass or in evaluating intracranial extension of a mass originating on the roof of the nasal cavity [6, 13, 21].T2-weighted MRI will reveal characteristic findings for a hemangioma: vascular tissue with multiple flow voids surrounding an inner matrix of higher-intensity tumor [21]. In children, congenital malformations such as meningoceles, dermoid cysts, angiomatous polyp, schwannoma, angiofibroma,and gliomas should be considered in the differential diagnosis of an intranasal mass and can be differentiated by CT or MR imaging [16, 17].

In this case series, each patient underwent an in-office biopsy of her intranasal lesion. In each case, suspicion for angiofibroma or a lesion that extended intracranially such as a meningocele or meningomyelocele was sufficiently low that we felt a biopsy could be performed safely. In both instances, the biopsy established a previously uncertain diagnosis and resulted in minimal bleeding that was easily controlled in the office.

The recommended treatment of LCH in the nasal cavity is conservative local excision with cautery at the base of the tumor for hemostasis [7, 15].This technique is associated with low rates of recurrence [1, 7].Transnasal endoscopic resection is favored over a rhinotomy due to the relatively minimal morbidity of the procedure, the low rate of recurrence, and the better visualization of the tumor and the surrounding anatomy [13, 15, 16, 21].Our institution has previously demonstrated the utility of image guidance in sinonasal endoscopic surgery in the pediatric population [22].

Nasal LCH is a rare cause of an intranasal mass and is associated with unilateral epistaxis, nasal obstruction, epiphora, and purulent rhinorrhea.We advocate for image-guided endoscopic excision ofLCH in the adolescent population.

## Figures and Tables

**Table 1:** Pediatric lobular capillary hemangioma in the literature.

Study	Age (yrs)	Gender	Location in NC	Radiologic study	Histopathologic findings
Case 1	16	F	R middle turbinate	CT without contrast: mass in R NC.	Granulation tissue acutely inflamed. Dense meshwork of capillaries. LCH.

Case 2	12	F	L middle meatus	CT with contrast: mass in L NC	Proliferating small vessels in myxoid stroma; lobular architecture in some foci. Benign capillary hemangioma.

Burlucchi et al., 2010 [14]	5 mo	M	L inferior septum	MRI, T2 precontrast: heterogeneously hyperintense MRI, T1 postcontrast: marked though incomplete enhancement.	LCH

Benoit et al., 2010 [15]	5	M	R septum	Not specified	LCH

Puxeddu et al., 2006 [6]	∗	∗	∗	CT	LCH

Katori and Tsukuda, 2005 [16]	11	M	R lateral wall	CT: confirmed mass MRI, T1: mass isointense to muscle MRI, T2: hyperintense mass with small flow voids.	Keratinized squamous mucosa and lobules of capillaries in fibrous stroma. LCH.

Özcan et al., 2004 [13]	6	F	R floor	CT: soft tissue mass; no bony extension.	Lobules of dilated and congested capillaries with heavy inflammatory cell infiltration. LCH.

Karagama et al., 2002 [17]	8	M	L floor	None	Keratinized squamous mucosa and lobules of capillaries in fibrous stroma. LCH.

Kapella et al., 2001 [18]	7	F	L vestibule	CT	LCH

Simo et al., 1998 [11]	7	M	R lateral wall	Imaging obtained.	LCH

Stacey et al., 1980 [19]	10	F	Septum	None	LCH

∗: Not reported.
